# The Korea National Health and Nutrition Examination Survey data linked Cause of Death data

**DOI:** 10.4178/epih.e2022021

**Published:** 2022-02-09

**Authors:** Sungha Yun, Kyungwon Oh

**Affiliations:** Division of Health and Nutrition Survey and Analysis, Bureau of Chronic Disease Prevention and Control, Korea Disease Control and Prevention Agency, Cheongju, Korea

**Keywords:** Korea National Health and Nutrition Examination Survey, Cause of Death Statistics, Linkage data, Cause of death

## Abstract

The Korea National Health and Nutrition Examination Survey (KNHANES) is a national health survey that is conducted annually to assess the health and and health-related behaviors of Korean population. To utilize KNHANES data to studies of mortality risk factors, the Korea Disease Control and Prevention Agency (KDCA) constructed a database linking KNHANES data to cause-of-death statistics in Statistics Korea, made available to researchers since 2020. The KNHANES data were linked to the Cause of Death Statistics based on resident registration numbers for subjects aged 19 years or older who agreed to link the data. The linkage rate between 2007-2015 National Health and Nutrition Examination Survey and 2007-2019 Cause of Death Statistics was 97.1%. In the linked dataset, the total death rate was 6.6%, of which neoplasms accounted for the highest death rate (32.1%), followed by circulatory system disease (22.7%) and respiratory system disease (11.5%). The linked dataset was made available through the Research Data Center of the KDCA after a review of the research proposal, and will be made available after periodical updates.

## INTRODUCTION

The Korea Disease Control and Prevention Agency (KDCA) has annually conducted the Korea National Health and Nutrition Examination Survey (KNHANES) to assess the health and and health-related behaviors of Korean population based on article 16 of the National Health Promotion Act. The KNHANES is being used as the basis for health policy establishment and evaluation [1,2]. In addition, data from the KNHANES are made available to the public to be used as a data source for various epidemiologic studies. However, some limitations make it difficult to identify the directionality of the relationship between the health behaviors and nutritional status and chronic diseases due to the characteristics of a cross-sectional study.

To complement the limitations of a cross-sectional study and enhance the utilization of the survey data, other countries link national health survey data to healthcare service utilization and cause of death data, and use these linked datasets in various studies. In the United States, the National Health Interview Survey and the National Health and Nutrition Examination Survey provide researchers with the data linked to Medicare and Medicaid Services data, including healthcare service utilization-related information and the National Death Index data that includes causeof-death information [3,4]. In Australia, National Health Survey data are linked to Multi-Agency Data Integration Project data, and the linked dataset is used for studies on the relationship between health and healthcare services [5]. Similarly, data from the Canadian Community Health Survey have been linked to the Canadian Death Database, the Historical Tax Summary file, and the Longitudinal Immigration Database [6].

In Korea, the National Health Insurance Service (NHIS) and the Health Insureance Review and Assessment Service (HIRA) provides linked dataset on diseases, healthcare service utilization, and health examination information and the data collected by researchers upon their requests [7,8]. Also the Statistics Korea (KOSTAT) links and provides the Cause of Death Statistics and researcher’s data [9]. To increase the utilization of KNHANES data, the KDCA obtained consent from KNHANES participants to link the Cause of Death Statistics of KOSTAT, the Korea Central Cancer Registry of the National Cancer Center, and healthcare service utilization data of the NHIS, and the HIRA since 2007. Based on these data, the KDCA has established the KNHANES data linked the Cause of Death Statistics, which has been provided to researchers from 2020 onwards [10]. The present report introduces the composition of the linked dataset, major outcomes, and disclosure procedures.

## DATA RESOURCES

The KNHANES has been conducted to produce health statistics of Korean population aged 1 year or older. Sample design, subjects, survey components, and survey methods of the KNHANES are described in the Guidebook for Korea National Health and Nutrition Examination Survey database and related publications [1,2,11]. The KNHANES survey can be briefly summarized as follows (Table 1): To obtain the KNHANES samples, the most recent available data of the Population and Housing Census was used as a sampling frame at the time of the design, in which a stratified multistage probability sampling design with enumeration district, household, etc. as sampling units was used. Subjects for sampling include Korean population who were all family members aged 1 year or older in the selected primary sampling units and households, corresponding to about 10,000 individuals. The survey was composed of a health interview, a health examination survey, and a nutrition survey. In the health survey, household information such as household type, household income, etc., and personal health behavior such as smoking, alcohol use, physical activity, mental health, disease morbidity, etc. are collected by face to face interveiw or self-report method. The health examination survey was composed of body measurements, blood pressure, laboratory tests, etc., in which data were collected through measurements and examinations. In the nutrition survey, dietary behavior, daily food and dietary intake, food security of households, etc., are collected by face to face interveiw method. The health interview and the health examination survey were conducted in mobile examination centers, while the nutrition survey was performed by visiting households.

In accordance with article 18 of the Statistics Act, the Cause of Death Statistics are produced to provide fundamental data not only for the identification of numbers and causes of death among Korean population, but also for the establishment of healthcare policy. The Cause of Death Statistics are published the following year based on reports received from January of the current year to April of the following year, and consist of information on the date and time of death, cause of death, place of death, and residence area at the time of death. Causes of death were selected from underlying causes of death among those recorded on death certificates according to the International Classification of Diseases of the World Health Organization. These causes of death were then further classified following the Korean Standard Classification of Diseases, 7th revision [9].

Raw data from the KNHANES are released in December of the year following the survey, and the Cause of Death Statistics are disclosed during the first half of the following year of data collection. In the linked data, the KNHANES data is updated for each survey cycle by combining 3 years of data since the survey components of the KNHANES data are similar within a survey cycle (3 years). The Cause of Death Statistics are updated annually following the Cause of Death Statistics update interval (1 year) of KOSTAT.

In 2020, the linked dataset was released for the first time following the linkage of 2007-2015 KNHANES data to 2007-2018 Cause of Death Statistics (dataset version 1.1). The currently available dataset (dataset version 1.2) are the linkage of 2007-2015 KNHANES data to 2007-2019 Cause of Death Statistics. In 2022, both the KNHANES and the Cause of Death Statistics will be updated, at which time the linked dataset (dataset version 2.1) of 2007-2018 KNHANES data and 2007-2020 Cause of Death Statistics will be released.

## POPULATION COVERAGE

The KNHANES and the Cause of Death Statistics were linked based on resident registration numbers of participants that were collected from the KNHANES. Of the participants of the KNHANES health examination, participants aged 19 years or older who agreed to link their Cause of Death Statistics and had valid resident registration numbers were included in the KNHANES data linked Cause of Death Statistics (linked dataset). 53,101 people who were 19 years or older participated in the 2007-2015 KNHANES health examination survey (22,627 men and 30,474 women), of whom 98.9% (98.8% of men and 99.0% of women) consented to link their Cause of Death Statistics. 97.5% of these individuals (97.9% of men and 97.2% of women) had valid resident registration numbers. In total, 51,575 participants who agreed to link their data and had valid registration numbers were included in the linked dataset. The linkage rate was 97.1% (men 97.5%, women 96.8%) of all participants of the health examination survey (Table 2).

## MEASURES

The linked dataset contained all variables provided in the KNHANES raw data. In the linked dataset, since the age of death in the cause of death statistics is calculated based on resident registration, the age based on the actual date of birth was deleted and the age based on resident registration was additionally included. Also, the month of the health examination survey was added to calculate follow-up periods. Household and parental ID information, which was unable to be analyzed by the linked dataset, and survey items for children and youths were excluded. Death related information includeds the cause of death and the year and month of death. The causes of death in linked data are provided to researcher based on the subcategories of the Korean Standard Classification of Diseases (7th revision). However, certain infectious and parasitic diseases (A00-B99), mental and behavioral disorders (F00-F99), and external causes of morbidity and mortality (V01-Y98) were sensitive information, so subcategories of these causes of death is provided after review of the research proposal.

The currently available linked dataset (linked dataset version 1.2) have the following characteristics: when December 31, 2019, was selected as the cut-off date for the last day of follow-up, the mean follow-up period of 51,575 participants included in the linked dataset was 8.4 years, and the total sum of person-years (8.4× 51,575) was 419,628 person-years. Of the 51,575 participants included in the linked dataset, 3,426 died between 2007 and 2019 (6.6% in death rate). Death rates of men and women were 8.8% and 5.0%, respectively, indicating that men have a higher mortality rate than women. The death rate was higher when the participating age in the KNHANES was higher and when the income level was lower (Table 3). Since the linked dataset contained at least 97% of KNHANES participants, characteristics were not compared between those who were included or excluded from the linked dataset.

Of the main categories of causes of death, death due to neoplasms accounted for the highest proportion (32.1%), followed by diseases of the circulatory system (22.7%), diseases of the respiratory system (11.5%), external causes of morbidity and mortality (9.6%), and symptoms and signs not elsewhere classified (7.6%) (Table 4). While the leading causes of death were similar between men and women, the order was different. Men had the same results as the results for Korean population [12], whereas the leading causes of death for women were neoplasms, diseases of the circulatory system, symptoms and signs not elsewhere classified, diseases of the respiratory system, and external causes of morbidity and mortality, in that order. When the causes of death that showed high mortality were analyzed in greater detail, deaths due to malignant neoplasms of the trachea, bronchus, and lungs accounted for the highest proportion of deaths caused by neoplasms (7.5%), followed by malignant neoplasms of the liver and intrahepatic bile ducts (4.2%), malignant neoplasms of the stomach (3.9%), and malignant neoplasms of colon, rectum, and anus (3.0%). Among diseases of the circulatory system, deaths due to cerebrovascular diseases (8.3%) had the highest proportion, followed by ischemic heart diseases (6.0%), and other heart diseases (5.1%). Among diseases of the respiratory system, deaths due to pneumonia (6.1%) accounted for the highest proportion.

**Figure 1.**
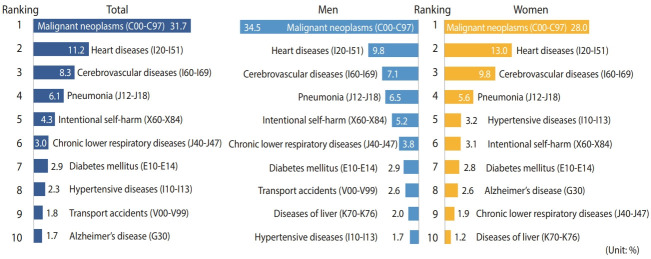
The 10 leading causes from 56 selected cause [12] of death among adults aged 19 years and over in the Korea National Health and Nutrition Examination Survey linked Cause of Death data (dataset version 1.2.).

Of the general death categories (56 items) of KOSTAT, the leading causes of death were malignant neoplasms (31.7%), heart diseases (11.1%), cerebrovascular diseases (8.3%), pneumonia (6.1%), and intentional self-harm (4.3%). These results are similar to those of the 2019 annual report on the Cause of Death Statistics, although there were differences in the order of the causes of death (Figure 1) [12].

## DATA RESOURCE UTILIZATION

To disclose the linked dataset, pilot studies and disclosure risk assessment for linked dataset were performed [13]. Based on the results of these studies, a disclosure procedure and a guidebook were prepared, and then the data were disclosed for the first time during the first half of 2020. Specific measures for utilization of the linked dataset were prepared through pilot studies (a total of 5), and 7 research papers were published, which described the risk of death depending on various risk factors such as smoking, nutrient intake, blood pressure, sleep, work hours, and heavy metals [14-20]. From the disclosure of the linked dataset in February 2020 to October 31, 2021, a total of 35 cases were provided and have been utilized for analyses of various research topics.

## STRENGTHS AND WEAKNESSES

By linking the Cause of Death Statistics to the KNHANES, it became possible to use the KNHANES, a cross-sectional survey, as a prospective follow-up survey. The KNHANES is the most indepth survey of national health in Korea and contains information concerning roughly 500 items related to socioeconomic status, health behaviors (smoking, alcohol use, physical activity, etc.), nutrition, and chronic disease status (obesity, hypertension, diabetes, pulmonary diseases, ocular diseases, etc.); thus, it is possible to analyze risk factors for various chronic diseases and deaths through the linked dataset described herein. The KNHANES has assured data quality through data collection by well-trained full-time field staffs, and internal and external (relevant academic societies) quality control of survey procedures. To supplement missing reports of death, the Cause of Death data additionally reflect data from a supplementary survey on causes of death (direct survey with medical institutions), infant cremation report data, and information about the deceased without family or friends, securing the inclusiveness of the collected data. The Causes of Death data were also reviewed to confirm reported causes of death by examining administrative data, and periodically analyzed logical errors and consistency, enhancing the data validity. Thus, the linked dataset can be considered to provide consistency and accuracy in terms of quantity and quality because of its basis on these two datasets. Additional strengths of the linked dataset include the maintained representativeness of the KNHANES samples due to the high linkage rate between the two datasets (97.1%), and high accuracy of data linkage due to construction based on resident registration numbers. Lastly, both datasets are collected annually and disclosed to the public, enabling timely updates and provision of the updated linked dataset according to the update cycle.

Limitations of the linked dataset are as follows: first, while main categories for causes of death can be analyzed, analyses of causes of death in intermediate categories and subcategories are limited because the follow-up period of the linked dataset was not long enough. It is expected that the data can be analyzed more in-depth with respect to various topics as the follow-up period increases in the future. Second, the KNHANES excluded those who are unable to move and those who reside in institutionalized settings such as hospitals, nursing homes, and care centers; therefore, the total number of deaths or death rates by cause of death might have been either overestimated or underestimated. Third, survey data including socioeconomic status, health-related behaviors, chronic disease having, etc., were collected as part of the KNHANES, and may be changed over the follow-up period. Thus, these limitations should be considered when interpreting the results. Lastly, the linked dataset is provided to researchers through the Research Data Center of the KDCA, which limits accessibility. To improve accessibility to this dataset, a remote analysis system will be established and operated in the future.

## DATA ACCESSIBILITY

The linked dataset was made available after reviewing the relevant research proposals. Here, we briefly explain the procedure for providing the linked dataset: a researcher first submits a research proposal to the KDCA for review and requests the KOSTAT Microdata Integrated Service system for linking to the Cause of Death data following review. The KDCA then sends the required KNHANES raw data to the KOSTAT, and KOSTAT links this to the appropriate the Cause of Death data. Thereafter, the KDCA uploads the linked dataset from KOSTAT to the Research Data Center. The researcher then visits the Research Data Center in the KDCA, performs the analysis, and submits a request to transfer the results out of the center. After reviewing the analysis results, the KDCA sends them to the researcher by e-mail. A detailed procedure is described in the ‘Guidebook for Korea National Health and Nutrition Examination Survey Linked Cause of Death data’ on the KNHANES homepage (https://knhanes.kdca.go.kr) [10].

## CONCLUSION

To address the limitations of the KNHANES cross-sectional study, the KDCA has linked KNHANES data to the Cause of Death Statistics of KOSTAT and then disclosed the linked dataset. This linked dataset will be used as the basis for health policies and has been made available for various areas of research such as studies on risk factors that influence morbidity and mortality. The currently available linked dataset can be used for analyses of some causes of death such as neoplasms, diseases of the circulatory system, and diseases of the respiratory system, but is limited because the survey follow-up period was not sufficiently long. However, the Cause of Death data and the KNHANES will be updated every 1 year and 3 years, respectively, which will enable more diverse and in-depth studies with longer follow-up periods.

### Ethics statement

The 2007-2014 KNHANES was approved by the Institutional Review Board (IRB) of the KDCA (2007-02CON-04-P, 2008-04EXP-01-C 2009-01CON-03-2C 010-02CON-21-C 2011-02CON06-C 2012-01EXP-01-2C 2013-07CON-03-4C 2013-12EXP-03-5C), whereas the review was waived in 2015 according to Article 1 (1) of the Bioethics and Safety Act and Article 2 (2)-1 of the Enforcement Decree of the Bioethics and Safety Act. The linkage data were constructed only for participants who agreed to link the Cause-of-Death Statistics and provide it to the third parties, and then they are provided to researchers who had the IRB approval for the research proposal.

## Figures and Tables

**Table 1.** Summary of the Korea National Health and Nutrition Examination Survey and the Cause of Death Statistics

Items			Contents
Korea National Health and Nutrition Examination Survey			
	Subject	2007-2009	200 Primary sampling units, about 4,600 households, about 10,000 persons
		2010-2015	192 Primary sampling units, about 3,840 households, about 10,000 persons
	Survey components	Health interview	Socioeconomic status, smoking, alcohol use, physical activity, mental health, quality of life, chronic disease status, etc.
		Health examination	Body measurements, blood pressure, laboratory test, spirometry, dental caries, vision, retinal photo and visual field, audiometry, balance, etc.
		Nutrition survey	Food and dietary intake, dietary behavior, dietary supplement use, food security, etc.
	Survey methods	Health interview	Face-to-face interview or self-administered in the mobile examination center
		Health examination	Measurement and examination in the mobile examination center
		Nutrition survey	Face-to-face interview in sample person’s home
	Indicators	About 250 health indicators regarding health behaviors, nutrition status, chronic disease conditions	
Cause of Death Statistics			
	Subject	All Korean population who resided in Korea	
	Survey methods	Collection of administrative data (death notification filed at local administration offices and death certificates issued by physicians)	
	Indicators	Dates of death, cause of death, place of death, place of residence	

**Table 2.** Linkage information between 2007-2015 Korea National Health and Nutrition Examination Survey (KNHANES) and 2007-2019 Cause of Death Statistics (dataset version 1.2.)

Gender	Participants aged 19 yr and over in the examination survey of 2007-2015 KNHANES^1^	Participants agreed to link to the Cause of Death Statistics	Participants with valid resident registration	Participants linked to the Cause of Death Statistics^2^
Total	53,101	52,523 (98.9)	51,770 (97.5)	51,575 (97.1)
Men	22,627	22,366 (98.8)	22,160 (97.9)	22,070 (97.5)
Women	30,474	30,157 (99.0)	29,610 (97.2)	29,505 (96.8)

Values are presented as number or number (%).

1 Age calculated based on the resident registration number, which is different from the age reported in the KNHANES that is calculated based on the actual date of birth.

2 Participants aged 19 years and over in the examination survey of the 2007-2015 KNHAHES who agreed to link their data to Cause of Death Statistics and obtained a valid resident registration number.

**Table 3.** Number and percentage of deaths among adults aged 19 years and over in the Korea National Health and Nutrition Examination Survey linked to Cause of Death data (dataset version 1.2.)

Variables		Total		Men		Women	
		n	Death, n (%)	n	Death, n (%)	n	Death, n (%)
Total		51,575	3,426 (6.6)	22,070	1,949 (8.8)	29,505	1,477 (5.0)
Survey year							
	2007	2,916	324 (11.1)	1,230	181 (14.7)	1,686	143 (8.5)
	2008	6,674	715 (10.7)	2,797	371 (13.3)	3,877	344 (8.9)
	2009	7,341	636 (8.7)	3,184	352 (11.1)	4,157	284 (6.8)
	2010	6,103	430 (7.0)	2,647	257 (9.7)	3,456	173 (5.0)
	2011	5,976	395 (6.6)	2,559	234 (9.1)	3,417	161 (4.7)
	2012	5,845	315 (5.4)	2,447	189 (7.7)	3,398	126 (3.7)
	2013	5,655	242 (4.3)	2,442	147 (6.0)	3,213	95 (3.0)
	2014	5,563	215 (3.9)	2,348	115 (4.9)	3,215	100 (3.1)
	2015	5,502	154 (2.8)	2,416	103 (4.3)	3,086	51 (1.7)
Age at survey^1^							
	19-29	6,313	25 (0.4)	2,729	19 (0.7)	3,584	6 (0.2)
	30-39	9,562	63 (0.7)	3,994	36 (0.9)	5,568	27 (0.5)
	40-49	9,717	140 (1.4)	4,221	85 (2.0)	5,496	55 (1.0)
	50-59	9,606	296 (3.1)	4,109	195 (4.8)	5,497	101 (1.8)
	60-69	8,469	697 (8.2)	3,794	455 (12.0)	4,675	242 (5.2)
	70-79	6,333	1,433 (22.6)	2,667	831 (31.2)	3,666	602 (16.4)
	≥80	1,575	772 (49.0)	556	328 (59.0)	1,019	444 (43.6)
Income level at survey^2^							
	Low	10,117	880 (8.7)	4,336	524 (12.1)	5,781	356 (6.2)
	Middle low	10,198	671 (6.6)	4,377	398 (9.1)	5,821	273 (4.7)
	Middle	10,135	606 (6.0)	4,347	345 (7.9)	5,788	261 (4.5)
	Middle high	10,174	601 (5.9)	4,327	329 (7.6)	5,847	272 (4.7)
	High	10,136	551 (5.4)	4,354	297 (6.8)	5,782	254 (4.4)

1 Age calculated based on the resident registration number.

2 Equivalent income of household=monthly household income/√No. of a household member.

**Table 4.** The distribution of cause of death among adults aged 19 years in the Korea National Health and Nutrition Examination Survey linked to Cause of Death data (dataset version 1.2.)

Cause of death^1^		Total (n=3,426)	Men (n=1,949)	Women (n=1,477)
Certain infectious and parasitic diseases (A00-B99)		85 (2.5)	40 (2.1)	45 (3.0)
Neoplasms (C00-D48)		1,101 (32.1)	679 (34.8)	422 (28.6)
	Malignant neoplasms of trachea, bronchus and lung (C33-C34)	258 (7.5)	193 (9.9)	65 (4.4)
	Malignant neoplasm of liver and intrahepatic bile ducts (C22)	145 (4.2)	102 (5.2)	43 (2.9)
	Malignant neoplasm of stomach (C16)	133 (3.9)	82 (4.2)	51 (3.5)
	Malignant neoplasms of colon, rectum and anus (C18-C21)	102 (3.0)	61 (3.1)	41 (2.8)
	Other neoplasms (Re. C00-D48)	463 (13.5)	241 (12.4)	222 (15.0)
Diseases of the blood & blood-forming organs (D50-D89)		12 (0.4)	4 (0.2)	8 (0.5)
Endocrine, nutritional and metabolic diseases (E00-E88)		115 (3.4)	71 (3.6)	44 (3.0)
Mental and behavioral disorders (F01-F99)		37 (1.1)	21 (1.1)	16 (1.1)
Diseases of the nervous system (G00-G98)		109 (3.2)	47 (2.4)	62 (4.2)
Diseases of the circulatory system (I00-I99)		778 (22.7)	383 (19.7)	395 (26.7)
	Cerebrovascular diseases (I60-I69)	283 (8.3)	138 (7.1)	145 (9.8)
	Ischemic heart diseases (I20-I25)	207 (6.0)	115 (5.9)	92 (6.2)
	Other heart diseases (I26-I51)	175 (5.1)	75 (3.8)	100 (6.8)
	Other diseases of the circulatory system (Re. I00-I99)	113 (3.3)	55 (2.8)	58 (3.9)
Diseases of the respiratory system (J00-J98, U04)		393 (11.5)	253 (13.0)	140 (9.5)
	Pneumonia (J12-J18)	208 (6.1)	126 (6.5)	82 (5.6)
	Other diseases of the respiratory system (Re. J00-J98, U04)	185 (5.4)	127 (6.5)	58 (3.9)
Diseases of the digestive system (K00-K92)		113 (3.3)	72 (3.7)	41 (2.8)
Diseases of the skin and subcutaneous tissue (L00-L98)		2 (0.1)	1 (0.1)	1 (0.1)
Diseases of the musculoskeletal and connective tissue (M00-M99)		13 (0.4)	2 (0.1)	11 (0.7)
Diseases of the genitourinary system (N00-N98)		75 (2.2)	27 (1.4)	48 (3.2)
Congenital malformations and chromosomal abnormalities (Q00-Q99)		2 (0.1)	2 (0.1)	0 (0.0)
Symptoms and signs, NEC (R00-R99)		261 (7.6)	118 (6.1)	143 (9.7)
External causes of mortality (V01-Y98)		330 (9.6)	229 (11.7)	101 (6.8)
	Intentional self-harm (X60-X84)	147 (4.3)	101 (5.2)	46 (3.1)
	Other external causes (Re. V01-Y98)	183 (5.3)	128 (6.5)	55 (3.7)

Values are presented as number (%).

1 Korean Standard Classification of Diseases code (7th revision).

## SUPPLEMENTARY MATERIALS

Korean version is available at https://www.e-epih.org/.
