# Supplementary material for: The Korea National Health and Nutrition Examination Survey data linked Cause of Death data
Source: Epidemiol Health. 2022 Feb 9;44:e2022021. doi: 10.4178/epih.e2022021 (PMC9117103; doi:10.4178/epih.e2022021)
Supplement: Supplementary file 1 [file epih-44-e2022021-suppl.docx]

**Title: 국민건강영양조사 - 사망원인통계 연계자료 구축**

**The Korea National Health and Nutrition Examination Survey data linked Cause of Death data**

**Running Title: KNHANES linked Cause of Death data**

Sungha Yun, Kyungwon Oh

Division of Health and Nutrition Survey and Analysis, Bureau of Chronic Disease Prevention and Control, Korea Disease Control and Prevention Agency, Chungcheongbuk-do, Republic of Korea

Corresponding Author: Kyungwon Oh, PhD

Division of Health and Nutrition Survey and Analysis, Bureau of Chronic Disease Prevention and Control, Korea Disease Control and Prevention Agency, 187 Osongsaengmyeong 2-ro, Heungduk-gu, Cheongju 28159, Korea

E-mail: kwoh27@korea.kr

**ABSTRACT**

국민건강영양조사는 우리 국민의 건강과 영양 수준을 파악하기 위해 매년 실시하는 국가 단위 건강조사로 질병관리청에서는 국민건강영양조사가 사망위험요인 규명에 활용되도록 통계청의 사망원인통계와 연계자료를 구축하여 2020년부터 연구자에게 공개하고 있다. 자료연계는 국민건강영양조사 검진조사 참여자 중 통계청의 사망원인통계 연계에 동의한 만19세 이상을 대상으로 주민등록번호를 기반으로 실시하였다. 2007~2015년 국민건강영양조사와 2007~2019년 사망원인통계의 연계율은 97.1%이었다. 2007~2015년 국민건강영양조사와 2007~2019년 사망원인통계 연계자료 (이하 연계자료 1.2) 기준 총 사망률은 6.6%이었고, 신생물로 인한 사망이 32.1%로 가장 비율이 높았고 순환계통 질환(22.7%), 호흡계통 질환(11.5%) 순이었다. 연계자료는 연구계획 심의 후 질병관리청 내 학술연구자료처리실를 통해 제공되고 있으며, 주기적으로 갱신하여 재 공개할 계획이다.

**주제어:** 국민건강영양조사, 사망원인통계, 연계자료, 사망원인

**INTRODUCTION**

국민건강영양조사는 국민건강증진법 제16조에 근거하여 우리 국민의 건강 및 영양상태를 파악하기 위해 질병관리청에서 매년 실시하고 있으며, 건강행태, 식품 및 영양소 섭취, 만성질환 유병 및 관리 수준에 관한 국가 단위의 건강 통계 및 건강정책 수립 및 평가에 필요한 근거자료를 생산하고 있다 [1, 2]. 또한 국민건강영양조사 원시자료는 대국민에게 공개되어 다양한 역학연구의 자료원으로 활용되고 있다. 그러나 단면조사의 특성으로 인해 건강 및 영양상태와 만성질환과의 선후관계 파악이 어려운 제한점이 있다.

국외의 국가단위 건강조사는 단면조사의 제한점을 보완하고 조사자료의 활용도 증대를 위하여 의료이용, 사망원인 자료 등과 연계되어 다양한 연구 활용되고 있다. 미국의 National Health Interview Survey와 National Health and Nutrition Examination Survey는 의료이용 관련 정보가 포함된 Medicare and Medicaid Services 자료, 사망 정보가 포함된 National Death Index 자료와 연계되어 연구자에게 공개되고 있고[3, 4]. 호주의 National Health Survey는 Multi-Agency Data Integration Project 자료와 연계되어 건강행태 및 수준과 의료 서비스와의 관련성에 대한 연구에 활용되고 있다[5]. 캐나다의 Canadian Community Health Survey도 Canadian Mortality Database, Historical Tax Summary file, Longitudinal Immigration Database와 연계되어 활용되고 있다[6].

우리나라는 국민건강보험공단과 건강보험심사평가원에서 연구자가 요청 시 상병, 의료이용, 건강검진 정보 등의 자료를 연구자가 수집한 자료와 연계하여 제공하고 있고[7, 8], 통계청에서는 사망원인통계와 연계·제공하고 있다[9]. 질병관리청은 국민건강영양조사 자료의 활용도 제고를 위해 2007년부터 국민건강영양조사에 참여한 대상자로부터 통계청의 사망원인통계, 국립암센터의 중앙암등록자료, 국민건강보험공단 및 건강보험심사평가원의 의료이용 자료와의 연계에 관한 동의를 구득하고 있고, 이를 기반으로 2020년부터 사망원인통계와 연계 자료를 구축하여 연구자에게 공개하고 있다 [10]. 본 연구에서는 국민건강영양조사-사망원인통계 연계자료의 구성, 주요결과 및 공개절차 등에 대해 간략하게 소개하고자 한다.

**DATA RESOURCES**

국민건강영양조사는 우리나라에 거주하는 만1세이상 국민의 대표성있는 건강통계 생산을 목표로 수행되고 있다. 국민건강영양조사의 표본설계, 조사대상, 조사항목, 조사방법 등은 국민건강통계, 원시자료 이용지침서, 관련 논문 등에 자세하게 기술되어 있다[1, 2, 11]. 국민건강영양조사 조사체계를 간략하게 기술하면 다음과 같다(표 1). 국민건강영양조사 표본은 설계시점에서 가용 가능한 가장 최근의 인구주택총조사 자료를 추출틀로 활용, 조사구, 가구 등을 추출단위로 하는 층화집락표본추출방법을 이용하여 추출하고 있다. 조사대상은 표본으로 추출된 조사지역 내 대상 가구의 만1세 이상의 모든 가구원, 약 10,000명이다. 조사는 건강설문조사, 영양조사, 검진조사로 구성되어 있다. 건강설문조사에서는 가구유형, 가구소득 등의 가구 정보와 흡연, 음주, 신체활동, 정신건강, 질병이환 등의 개인별 건강행태에 대해 면접 또는 자기기입 방법으로 조사하고 있다. 영양조사에서는 식생활 행태, 1일 동안의 식품 섭취, 가구의 식품안정성 등에 관하여 면접방법으로 조사하고 있다. 검진조사는 신체계측, 혈압측정, 임상검사 등으로 구성되어 있으며 계측 및 검진을 통해 자료를 수집하고 있다. 건강설문조사와 검진조사는 이동검진차량에서, 영양조사는 대상가구를 방문하여 조사를 실시하고 있다.

사망원인통계는 통계법 제18조에 의거하여 우리 국민의 사망 규모와 원인 파악 및 보건의료 정책 수립을 위한 기초자료 제공을 목적으로 생산되고 있다. 사망원인통계는 당해년도 1월부터 익년 4월까지 접수된 신고서 중 당해년도 사망자를 집계하여 익년에 발표하고 있으며, 사망 일시, 사망 원인, 사망 장소, 사망 시 거주지역 등에 관한 정보가 포함되어 있다. 사망원인은 세계보건기구의 국제표준질병사인분류지침에 따라 사망진단서의 기재 사인 중 원사인(underlying cause of death)을 선정하였으며, 한국표준질병사인분류(제7차 개정)에 따라 분류하고 있다 [9].

국민건강영양조사 원시자료의 경우 조사연도 익년 12월에 공개하고, 사망원인통계는 당해연도 익년 상반기에 공개하고 있다. 연계자료에서 국민건강영양조사 자료의 갱신은 조사항목이 기수 별로 유사하므로 기수 별(3년 단위)로 통합하여 갱신하고 있다. 사망원인통계는 통계청의 사망원인통계 갱신주기(1년)에 따라 매년 갱신하고 있다.

연계자료는 2007년~2015년 국민건강영양조사를 기반으로 2007년~2018년 사망원인통계와 연계(버전 1.1)하여 2020년 최초 공개되었으며, 현재 공개 중인 자료는 2007년~2015년 국민건강영양조사와 2007년~2019년 사망원인통계를 연계한 자료(버전 1.2)이다. 2022년에는 국민건강영양조사와 사망원인통계를 모두 갱신하여 2007년-2018년 국민건강영양조사와 2007년~2020년 사망원인통계 연계자료(버전 2.1)를 공개할 예정이다.

**POPULATION COVERAGE**

국민건강영양조사와 사망원인통계는 국민건강영양조사에서 수집한 참여자의 주민등록번호를 기반으로 연계하였다. 연계대상은 국민건강영양조사 검진조사 참여자 중 사망원인통계 연계에 동의하고 주민등록번호가 유효한 만19세 이상이었다. 2007~2015년 국민건강영양조사 만19세 이상 검진조사 참여자는 53,101명이었으며(남자 22,627명, 여자 30,474명), 이 중 98.9%(남자 98.8%, 여자 99.0%)가 사망원인통계 연계에 동의하였고, 주민등록번호가 유효한 분율은 97.5%(남자 97.9%, 여자 97.2%)이었다. 최종 연계대상은 51,575명으로 연계율은 검진조사 참여자를 기준으로 97.1%(남자 97.5%, 여자 96.8%)이었다(표 2).

**MEASURES**

연계자료에는 국민건강영양조사 원시자료로 제공되고 있는 모든 변수가 포함되어있다. 또한 사망원인통계와 주민등록번호를 기반으로 연계하고 사망원인통계의 사망연령이 주민등록기반으로 산출하므로 주민등록번호 기반 만나이가 포함되어있으며, 추적기간을 산출하기 위해 검진조사 월이 추가되어있고, 연계자료로 분석이 불가한 가구 및 부모 ID, 소아, 청소년 대상 조사 항목은 제외되어있다. 사망에 대한 정보는 사망여부, 사망원인, 사망 년, 월이 포함되어있다. 사망원인은 한국표준질병사인분류(제7차 개정)의 소분류를 기반으로 제공하되 특정 감염성 및 기생충성 질환(A00-B99), 정신행동장애(F00-F99), 질병이환 및 사망의 외인(V01-Y98)은 민감사인으로 대분류로 제공하고 있으며 민감사인의 소분류는 연구자의 연구계획에 따라 필요하다고 판단될 경우 제공하고 있다. 연계자료로 분석이 불가한 가구 및 부모 ID, 소아, 청소년 대상 조사 항목은 제외되어있다.

현재 공개 중인 국민건강영양조사와 사망원인통계 연계자료(버전 1.2)의 특성은 다음과 같다. 추적종료일을 2019년 12월 31일로 하였을 때 연계자료 대상자 51,575명의 평균 추적기간은 8.4년이고 총 추적 인년의 합은 419,628인년이었다. 연계대상자 51,575명 중 2007년~2019년 사망자는 3,426명으로 사망률은 6.6%이었다. 사망률은 남자 8.8%, 여자 5.0%로 남자가 여자보다 높았으며, 국민건강영양조사 참여 당시의 나이가 많을수록, 소득수준이 낮을수록 높은 경향이었다(표 3). 자료의 연계율이 97% 이상이므로 연계 대상자와 비 대상자의 특성은 추가로 비교하지 않았다.

사망원인별 분포를 대분류 기준으로 살펴보면, 신생물로 인한 사망 분율이 32.1%로 가장 높았고 그 다음으로 순환계통의 질환(22.7%), 호흡계통의 질환(11.5%), 질병이환 및 사망의 외인(9.6%), 달리 분류되지 않은 증상, 징후(7.6%)순이었다(표 4). 상위 분포를 차지하는 사망원인은 남녀가 동일했으나 순위에는 차이가 있었다. 남자의 경우 전체 대상자의 결과와 동일했으나, 여자의 경우 신생물, 순환계통 질환 다음으로 달리 분류되지 않은 증상, 징후로 인한 사망 분율이 높았고, 그 다음으로 호흡계통의 질환, 질병이환 및 사망의 외인 순이었다. 높은 분율을 나타냈던 사망원인에 대해 중분류로 좀 더 자세히 살펴보면, 신생물로 인한 사망 중에는 기관, 기관지 및 폐의 악성신생물로 인한 사망이 7.5%로 가장 높았고, 그 다음으로 간 및 간내 담관의 악성신생물(4.2%), 위의 악성신생물(3.9%), 결장, 직장 및 항문의 악성신생물(3.0%) 순이었다. 순환계통 질환 중에서는 뇌혈관질환으로 인한 사망(8.3%)이 가장 높았고 그 다음으로 허혈성 심장질환(6.0%), 기타 심장질환(5.1%) 순이었다. 호흡계통 질환 중에서는 폐렴으로 인한 사망(6.1%)이 가장 비율이 높았다.

통계청 일반사망선택분류표(56개 항목)에 따른 사망원인 순위를 살펴보면, 악성신생물(31.7%), 심장질환(11.1%), 뇌혈관질환(8.3%), 폐렴(6.1%), 고의적 자해(4.3%)가 상위를 차지하고 있었고 통계청 2019년 사망원인통계와 일부 사망원인의 순위 차이는 있으나 유사하였다 (그림 1) [12].

**DATA RESOURCES USE**

연계자료 공개를 위해 시범 연구, 개인정보 영향 평가를 실시하였고[13] 이를 기초로 연계방안 및 이용지침서를 마련한 후 2020년 상반기에 처음으로 공개하였다. 시범 연구(총5개)를 통해 연계자료의 구체적인 활용방안을 마련하였으며, 흡연, 영양소 섭취, 혈압, 수면, 노동시간, 중금속 등 다양한 위험요인에 따른 사망 위험도에 관한 7편의 학술논문이 발표되었다[14-20]. 연계자료가 공개 된 2020년 2월부터 2021년 10월 31일까지 총 35건이 제공되었으며 다양한 연구주제 분석에 활용되고 있다.

**STRENGTHS and WEAKNESSES**

국민건강영양조사에 사망원인통계를 연계함으로 단면조사인 국민건강영양조사가 전향적 추적조사의 형태로 활용이 가능하게 되었다. 국민건강영양조사는 국가 단위의 건강에 관한 가장 포괄적인 조사로 사회경제위치, 건강행태(흡연, 음주, 신체활동 등), 영양, 만성질환(비만, 고혈압, 당뇨병, 폐질환, 안질환 등)에 관한 500여개 항목에 관한 정보가 포함되어있으므로 연계자료를 통해 다양한 만성질환 및 사망의 위험요인에 대한 분석이 가능하다. 국민건강영양조사는 조사를 전담하는 조사원(전문조사수행팀)에 의한 자료 수집, 조사 수행 과정에 관한 내부, 외부(관련 학회) 질 관리를 실시하여 자료의 질을 확보하고 있다. 사망원인통계는 사망 신고의 누락을 보완하기 위해 사망원인보완조사(의료기관 대상 직접 조사), 영아 화장신고자료, 무연고 사망자 정보를 추가로 반영하여 수집 자료의 포괄성을 확보하고 있고, 행정자료를 이용하여 사망원인을 재확인하고 논리적 오류 및 정합성 분석을 주기적으로 실시하여 자료의 타당성을 확보하고 있다. 따라서 연계자료는 두 자료를 기반으로 구축되었으므로 양과 질이 확보된 자료라고 할 수 있다. 또한 국민건강영양조사와 사망원인통계의 연계율(97.1%)이 매우 높아 국민건강영양조사 표본의 대표성이 유지되었으며 주민등록번호를 기반으로 구축되어 자료 연계의 정확도가 높은 점도 강점이라고 할 수 있다. 마지막으로 두 자료 모두 매년 조사 및 공개되고 있어 연계자료 갱신주기에 맞춰 시의성 있게 갱신 및 제공될 수 있다.

본 연계자료의 제한점은 다음과 같다. 첫째, 연계자료의 추적기간이 충분하지 않아 사망원인의 대분류에 따른 분석은 가능하나 사망원인의 중분류, 소분류에 따른 분석은 제한적일 수 있다. 향후 추적기간이 길어짐에 따라 다양한 주제에 대한 심층적인 분석이 가능할 것으로 기대하고 있다. 둘째, 국민건강영양조사는 거동불능 및 병원입원과 양로원, 요양원 등 시설에 거주하는 경우 조사대상에서 제외되므로, 총 사망 또는 사망원인별 사망률이 과대 또는 과소 평가될 가능성이 있다. 셋째, 사회경제위치, 건강행태, 만성질환 유병 등 조사자료가 국민건강영양조사 참여 당시 기준으로 수집되어 추적기간 동안 변화될 가능성이 있으므로 결과 해석 시 이에 관한 고려가 필요하다. 마지막으로 연계자료가 질병관리청 내 학술자료처리실을 통해 연구자에게 제공하여 접근성이 낮은 제한점이 있으며, 이를 개선하기 위해 향후 원격분석시스템을 구축, 운영할 계획이다.

**DATA ACCESSIBILITY**

연계자료는 연구자의 연구계획 심의 후 제공하고 있다. 연계자료 제공 절차를 간략하게 설명하면, 연구자는 질병관리청에 연구계획 심의를 신청하여 심의 절차를 거친 후 통계청 Microdata Integrated Service(MDIS) 시스템에 사망원인통계와 연계를 요청한다. 질병관리청은 연계에 필요한 국민건강영양조사 원시자료를 통계청에 보내고, 통계청은 국민건강영양조사와 사망원인통계를 연계한다. 이후 질병관리청은 통계청에서 받은 연계자료를 학술연구자료처리실에 업로드한다. 연구자는 질병관리청 내 학술연구자료처리실을 방문하여 분석을 실시한 후 결과 반출을 요청하면 질병관리청은 분석결과를 검토한 후 연구자에게 메일로 발송한다. 연계자료 이용에 대한 세부 절차는 국민건강영양조사 홈페이지의 ‘국민건강영양조사-사망원인통계 연계자료 이용지침서’에 상세하게 기술되어 있다 [10].

**CONCLUSION**

질병관리청은 국민건강영양조사의 단면조사로서의 제한점 보완 및 건강정책 근거 마련, 만성질환, 사망 관련 위험요인 규명 연구 등 다양한 분야에 활용될 수 있도록 통계청 사망원인통계와 연계하여 공개하고 있다. 현재 공개되고 있는 연계자료는 추적기간이 충분하지 않아 신생물, 순환계통 질환, 호흡계통 질환 등에 일부 사망원인 대해서만 분석만 가능하지만 향후 사망원인통계는 매년, 국민건강영양조사는 3년 주기로 갱신할 예정으로 다양하고 심층적인 연구가 가능할 것으로 기대한다.

**REFERENCE**

1. Oh K, Kim Y, Kweon S, Kim S, Yun S, Park S, Lee Y, Kim Y, Park O, Jeong K. Korea National Health and Nutrition Examination Survey, 20th anniversary: accomplishments and future directions. Epidemiol Health 2021;43:e2021025.

2. Korea Centers for Disease Control & Prevention Agency. Korea health statistics 2019: Korea National Health and Nutrition Examination Survey (KNHANES VII-3). Cheongju: 2020. [cited 2021 Aug 2]. <https://knhanes.kdca.go.kr/knhanes/sub04/sub04_04_01.do>.

3. Centers for Disease Control and Prevention. The linkage of National Center for Health Statistics Survey Data to the National Death Index-2019 Linked Mortality File (LMF): Linkage Methodology and Analytic Considerations. 2021. [cited 2021 Aug 2]. Available from <https://www.cdc.gov/nchs/data/datalinkage/2019NDI-Linkage-Methods-and-Analytic-Considerations-508.pdf>

4. Centers for Disease Control and Prevention. The Linkage of the 2016 National Hospital Care Survey to the 2016/2017 Centers for Medicare & Medicaid Services Medicare Enrollment, Claims/Encounters and Assessment Data: Matching Methodology and Analytic Considerations. 2020 [cited 2020 Sep 21]. Available from <https://www.cdc.gov/nchs/data/datalinkage/2016-nhcs-cms-linkage-methodology.pdf>.

5. Australian Bureau of Statistics. Independent Privacy Impact Assessment (PIA) on the National Health Survey (NHS) Linkage Project. 2018. [cited 2021 Aug 3]. Available from <https://www.abs.gov.au/websitedbs/d3310114.nsf/0/9099c77cb979d558ca258198001b27a0/$FILE/Linkage%20of%20National%20Health%20Survey%20with%20MADIP.pdf>

6. Statistics Canada. Linking the Canadian Community Health Survey and the Canadian Mortality Database: An enhanced data source for the study of mortality. 2016. [cited 2021 Aug 3]. Available from <https://www150.statcan.gc.ca/n1/en/pub/82-003-x/2016012/article/14687-eng.pdf?st=JSkcUgkl>

7. National Health Insurance Service. User manual of Sample cohort 2.0 database. 2017. [cited 2021 Aug 3]. Available from <https://nhiss.nhis.or.kr/bd/ab/bdaba005iv.do>

8. Health Insurance Review and Assessment Service. Guidebook for user of HIRA linkage data. 2021. [cited 2022 Mar 15]. Available from https://opendata.hira.or.kr/co.apndFile.dir/download.do?fileNm=%EA%B2%B0%ED%95%A9%EC%9E%90%EB%A3%8C_%EC%9D%B4%EC%9A%A9%EA%B0%80%EC%9D%B4%EB%93%9C.hwp

9. Statistics Korea. Statistics information report for Cause of death statistics. 2019. [cited 2021 Aug 3]. Available from <https://mdis.kostat.go.kr/infoData/detailData.do?statsConfmNo=101054>

10. Korea Centers for Disease Control & Prevention Agency. Guidebook for Korea National Health and Nutrition Examination Survey linked Cause of Death data. [cited 2021 Aug 3]. Available from <https://knhanes.kdca.go.kr/knhanes/sub09/sub09_01.do>

11. Korea Centers for Disease Control & Prevention Agency. Guidebook for Korea National Health and Nutrition Examination Survey database. 2016. [cited 2021 Aug 3]. Available from <https://knhanes.kdca.go.kr/knhanes/sub03/sub03_02_05.do>

12. Statistics Korea. Annual report on the Cause of Death Statistics. Daejeon: Statistics Korean; 2020. [cited 2021 Aug 3]. Available from <https://kosis.kr/publication/publicationThema.do?pubcode=YD>

13. Korea Centers for Disease Control & Prevention Agency. Final report for research program: Disclosure Risk Assessment for linking KNHANES microdata to Cause of Death microdata. Cheongju: Korea Centers for Disease Control & Prevention Agency; 2020. [cited 2022 Mar 15]. Available from <https://www.prism.go.kr/homepage/entire/downloadResearchAttachFile.do?workKey=001&fileType=CPR&seqNo=001&pdfConvYn=N&researchId=1351000-201900399>

14. Park YS, Park S, Lee C. The Attributable Risk of Smoking on All-Cause Mortality in Korean: A Study Using KNHANES IV–VI (2007–2015) with Mortality Data. Tuberc Respir Dis(Seoul) 2020;83:268-275

15. Park CY, Jo G, Lee J, Shigh GM, Lee J, Shin M. Association between dietary sodium intake and disease burden and mortality in Koreans between 1998 and 2016: The Korea National Health and Nutrition Examination Survey. Nutr Res Pract 2020;14(5):501-518

16. Yun D, Lee H, Choi W, Chang H, Son D, Lee J. Association of optimal blood pressure with mortality in patients taking antihypertensive medications. J Clin Hypertens 2020;00:1–9.

17. Kwon S, Lee H, Lee J, Shin M, Choi S, Oh H. Sleep duration and mortality in Korean adults: a population-based prospective cohort study. BMC Public Health 2020;20:1623-1633.

18. Lee H, Kim I, Kim H, Kawachi I. Association of long working hours with accidents and suicide mortality in Korea. Scand J Work Environ Health 2020;46(5):480–487.

19. Byun G, Lim S, Kim S, Park D, Shin M, Oh H, Lee J. Blood Lead Concentrations and Mortality in Korean Adults: the Korea National Health and Nutrition Examination Survey with Mortality Follow-Up. Int. J. Environ. Res. Public Health 2020;17:6898-6910.

20. Lee DH, Nam JY, Kwon S, Keum N, Lee J, Shin M, Oh H. Lifestyle risk score and mortality in Korean adults: a population-based cohort study. Scientific Reports 2020;10:10260

Table 1. Summary of the Korea National Health and Nutrition Examination Survey and the Cause of Death Statistics

|  | Contents | |
| --- | --- | --- |
| Korea National Health and Nutrition Examination Survey | | |
| Subject | 2007-2009 | 200 Primary sampling unit, about 4,600 household, about 10,000 sample person |
|  | 2010-2015 | 192 Primary sampling unit, about 3,840 household, about 10,000 sample person |
| Survey | Health interview | Socioeconomic status, smoking, alcohol use, physical activity, mental health, quality of life, chronic disease status, etc. |
| components | Health examination | Body measurements, blood pressure, laboratory test, spirometry, dental caries, vision, retinal photo and visual field, audiometry, balance, etc. |
|  | Nutrition survey | Food and dietary intake, dietary behavior, dietary supplement use, food security, etc. |
| Survey | Health interview | Face-to-face interview or self-administered in the mobile examination center |
| methods | Health examination | Measurement and examination in the mobile examination center |
|  | Nutrition survey | Face-to-face interview in sample person’s home |
| Indicators | About 250 health indicators in health behaviors, nutrition status, chronic disease conditions | |
| Cause of Death Statistics | | |
| Subject | All Koreans who had resided in Korea | |
| Survey | Collection of Administrative data (death notification filed at local administration offices and death medical certificates issued by physicians) | |
| methods |  |  |
| Indicators | Dates of death, cause of death, place of death, place of residence | |

Table 2. Linkage information between 2007-2015 Korea National Health and Nutrition Examination Survey (KNHANES) and 2007-2019 Cause-of-Death Statistics (dataset version 1.2.)

|  | Participants aged 19 yr and over in the examination survey of 2007-2015 KNHANES^1^ | Participants agreed to link to the Cause of Death Statistics | Participants with valid resident registration | Participants linked to the Cause of Death Statistics^2^ |
| --- | --- | --- | --- | --- |
| Total | 53,101 | 52,523 (98.9) | 51,770 (97.5) | 51,575 (97.1) |
| Men | 22,627 | 22,366 (98.8) | 22,160 (97.9) | 22,070 (97.5) |
| Women | 30,474 | 30,157 (99.0) | 29,610 (97.2) | 29,505 (96.8) |

Values are presented as number or number (%)

^1^Age calculated based on the resident registration number, which is different from the age reported in the KNHANES that is calculated based on the actual date of birth.

^2^Participants aged 19 years and over in the examination survey of the 2007-2015 KNHAHES who agreed to link their data to Cause of Death Statistics and obtained a valid resident registration number.

Table 3. Number and percentage of deaths among adults aged 19 years and over in the Korea National Health and Nutrition Examination Survey linked to Cause of Death data (dataset version 1.2.)

| Variables | **Total** | | |  | **Men** | | |  | **Women** | | |
| --- | --- | --- | --- | --- | --- | --- | --- | --- | --- | --- | --- |
|  | **n** | **Death, n (%)** | |  | **n** | **Death, n (%)** | |  | **n** | **Death, n (%)** | |
| Total | 51,575 | 3,426 | (6.6) |  | 22,070 | 1949 | (8.8) |  | 29,505 | 1,477 | (5.0) |
|  |  |  |  |  |  |  |  |  |  |  |  |
| Survey year | | | | | | | | | | | |
| 2007 | 2,916 | 324 | (11.1) |  | 1,230 | 181 | (14.7) |  | 1,686 | 143 | (8.5) |
| 2008 | 6,674 | 715 | (10.7) |  | 2,797 | 371 | (13.3) |  | 3,877 | 344 | (8.9) |
| 2009 | 7,341 | 636 | (8.7) |  | 3,184 | 352 | (11.1) |  | 4,157 | 284 | (6.8) |
| 2010 | 6,103 | 430 | (7.1) |  | 2,647 | 257 | (9.7) |  | 3,456 | 173 | (5.0) |
| 2011 | 5,976 | 395 | (6.6) |  | 2,559 | 234 | (9.1) |  | 3,417 | 161 | (4.7) |
| 2012 | 5,845 | 315 | (5.4) |  | 2,447 | 189 | (7.7) |  | 3,398 | 126 | (3.7) |
| 2013 | 5,655 | 242 | (4.3) |  | 2,442 | 147 | (6.0) |  | 3,213 | 95 | (3.0) |
| 2014 | 5,563 | 215 | (3.9) |  | 2,348 | 115 | (4.9) |  | 3,215 | 100 | (3.1) |
| 2015 | 5,502 | 154 | (2.8) |  | 2,416 | 103 | (4.3) |  | 3,086 | 51 | (1.7) |
|  |  |  |  |  |  |  |  |  |  |  |  |
| Age at survey^1^ | | | | | | | | | | | |
| 19-29 | 6,313 | 25 | (0.4) |  | 2,729 | 19 | (0.7) |  | 3,584 | 6 | (0.2) |
| 30-39 | 9,562 | 63 | (0.7) |  | 3,994 | 36 | (0.9) |  | 5,568 | 27 | (0.5) |
| 40-49 | 9,717 | 140 | (1.4) |  | 4,221 | 85 | (2.0) |  | 5,496 | 55 | (1.0) |
| 50-59 | 9,606 | 296 | (3.1) |  | 4,109 | 195 | (4.8) |  | 5,497 | 101 | (1.8) |
| 60-69 | 8,469 | 697 | (8.2) |  | 3,794 | 455 | (12.0) |  | 4,675 | 242 | (5.2) |
| 70-79 | 6,333 | 1,433 | (22.6) |  | 2,667 | 831 | (31.2) |  | 3,666 | 602 | (16.4) |
| 80+ | 1,575 | 772 | (49.0) |  | 556 | 328 | (59.0) |  | 1,019 | 444 | (43.6) |
|  |  |  |  |  |  |  |  |  |  |  |  |
| Income level at survey^2^ | | | | | | | | | | | |
| Low | 10,117 | 880 | (8.7) |  | 4,336 | 524 | (12.1) |  | 5,781 | 356 | (6.2) |
| Middle low | 10,198 | 671 | (6.6) |  | 4,377 | 398 | (9.1) |  | 5,821 | 273 | (4.7) |
| Middle | 10,135 | 606 | (6.0) |  | 4,347 | 345 | (7.9) |  | 5,788 | 261 | (4.5) |
| Middle high | 10,174 | 601 | (5.9) |  | 4,327 | 329 | (7.6) |  | 5,847 | 272 | (4.7) |
| High | 10,136 | 551 | (5.4) |  | 4,354 | 297 | (6.8) |  | 5,782 | 254 | (4.4) |

^1^ Age calculated based on the resident registration number.

^2^ Equivalent income of household=monthly household income/√No. of a household member.

Table 4. The distribution of cause of death among adults aged 19 years in the Korea National Health and Nutrition Examination Survey linked to Cause of Death data (dataset version 1.2.)

| **Cause of death^1^** | **Total**  **(n=3,426)** | | **Men**  **(n=1,949)** | | **Women**  **(n=1,477)** | |
| --- | --- | --- | --- | --- | --- | --- |
| Certain infectious and parasitic diseases (A00-B99) | 85 | (2.5) | 40 | (2.1) | 45 | (3.0) |
| Neoplasms(C00-D48) | 1,101 | (32.1) | 679 | (34.8) | 422 | (28.6) |
| Malignant neoplasms of trachea, bronchus and lung (C33-C34) | 258 | (7.5) | 193 | (9.9) | 65 | (4.4) |
| Malignant neoplasm of liver and intrahepatic bile ducts (C22) | 145 | (4.2) | 102 | (5.2) | 43 | (2.9) |
| Malignant neoplasm of stomach(C16) | 133 | (3.9) | 82 | (4.2) | 51 | (3.5) |
| Malignant neoplasms of colon, rectum and anus(C18-C21) | 102 | (3.0) | 61 | (3.1) | 41 | (2.8) |
| Other neoplasms(Re. C00- D48) | 463 | (13.5) | 241 | (12.4) | 222 | (15.0) |
| Diseases of the blood & blood-forming organs (D50-D89) | 12 | (0.4) | 4 | (0.2) | 8 | (0.5) |
| Endocrine, nutritional and metabolic diseases (E00-E88) | 115 | (3.4) | 71 | (3.6) | 44 | (3.0) |
| Mental and behavioral disorders (F01-F99) | 37 | (1.1) | 21 | (1.1) | 16 | (1.1) |
| Diseases of the nervous system (G00-G98) | 109 | (3.2) | 47 | (2.4) | 62 | (4.2) |
| Diseases of the circulatory system (I00-I99) | 778 | (22.7) | 383 | (19.7) | 395 | (26.7) |
| Cerebrovascular diseases (I60-I69) | 283 | (8.3) | 138 | (7.1) | 145 | (9.8) |
| Ischemic heart diseases (I20-I25) | 207 | (6.0) | 115 | (5.9) | 92 | (6.2) |
| Other heart diseases (I26-I51) | 175 | (5.1) | 75 | (3.8) | 100 | (6.8) |
| Other diseases of the circulatory system(Re. I00-I99) | 113 | (3.3) | 55 | (2.8) | 58 | (3.9) |
| Diseases of the respiratory system (J00-J98,U04) | 393 | (11.5) | 253 | (13.0) | 140 | (9.5) |
| Pneumonia (J12-J18) | 208 | (6.1) | 126 | (6.5) | 82 | (5.6) |
| Other diseases of the respiratory system(Re. J00-J98,U04) | 185 | (5.4) | 127 | (6.5) | 58 | (3.9) |
| Diseases of the digestive system (K00-K92) | 113 | (3.3) | 72 | (3.7) | 41 | (2.8) |
| Diseases of the skin and subcutaneous tissue (L00-L98) | 2 | (0.1) | 1 | (0.1) | 1 | (0.1) |
| Diseases of the musculoskeletal and connective tissue (M00-M99) | 13 | (0.4) | 2 | (0.1) | 11 | (0.7) |
| Diseases of the genitourinary system (N00-N98) | 75 | (2.2) | 27 | (1.4) | 48 | (3.2) |
| Congenital malformations and chromosomal abnormalities (Q00-Q99) | 2 | (0.1) | 2 | (0.1) | 0 | (0.0) |
| Symptoms and signs, NEC (R00-R99) | 261 | (7.6) | 118 | (6.1) | 143 | (9.7) |
| External causes of mortality (V01-Y98) | 330 | (9.6) | 229 | (11.7) | 101 | (6.8) |
| Intentional self-harm (X60-X84) | 147 | (4.3) | 101 | (5.2) | 46 | (3.1) |
| Other external causes(Re. V01-Y98) | 183 | (5.3) | 128 | (6.5) | 55 | (3.7) |

Values are presented as number (%)

^1^ Korean Standard Classification of Diseases code (7th Revision)


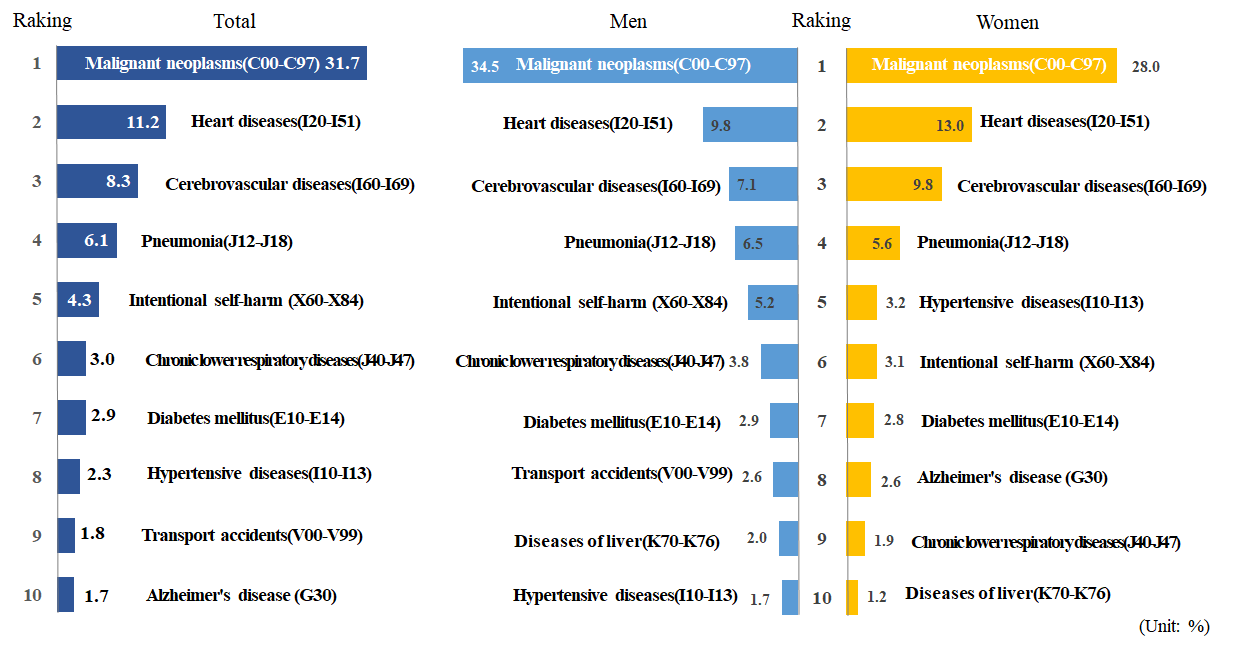


Figure 1. The 10 leading causes from 56 selected cause [12] of death among adults aged 19 years and over in the Korea National Health and Nutrition Examination Survey linked Cause of Death data (dataset version 1.2.)
